# U. S. P. 1890.—The Pharmacopœdia of the United States, Seventh Decennial Revision

**Published:** 1893-12

**Authors:** 


### Editorial, Etc.

### U. S. P. 1890.—THE PHARMACOPŒDIA OF THE UNITED STATES, SEVENTH DECENNIAL REVISION.

The new "U. S. P. 1890" will take the place of its predecessor, and become the official standard, on January 1st, 1894. The descriptions of official members of materia medica occupy 460 of the 600 pages of the volume, the remaining 200 pages being made up of a history of the work, a presentation of and arguments for the features of the book, and various tables of interest; these appendages might with advantage have been curtailed or omitted altogether. The official products are 994 in number, 2 less than in the 6th revision; 90 members of the 1880 U. S. P. were dropped, and 88 new products added. Of "new remedies" only a few have been included; all remedies "which cannot be produced otherwise than under patented process, or which are protected by proprietary rights" were excluded. This rule was disregarded in adding lanolin, salol, etc. Only three drugs, cinchona, nux vomica and opium, were standardized—i. e., require assayed strength. The metric system of weights and measures has been adopted and is now the sole standard. The nomenclature has not been materially changed.

The general rules governing the processes of manufacture, included in "Preliminary Notices" as a sort of preface, are not materially changed; in fact, for the greater part they are word for word identical with the text in the 1880 U. S. P. Only one revolutionizing change has been made, namely, in the system of weights and measures. The old and firmly intrenched Avoirdupois and Apothecaries weights, slighted ten years ago by the adoption of the compromise, half-way, unspecific and indefinite "parts by weight," have been entirely discarded now, and the metric system (*liquids* by volume, and *solids* by weight) substituted radically and without qualification. Although notice of this change was received three years ago when the Pharmacopœial Convention authorized this change, and despite the fact that our pharmaceutical journals have exerted themselves continuously to give their readers preliminary education in the metric system, the change will meet with much opposition, and it will take a long time to make its adoption general. The practice of a life time is not readily abandoned; and a few generations will have to pass away before the metric system can come into general use through the new recruits.

Optional permission is granted to add 10 per cent. glycerin to solid extracts which are to be kept in soft condition, suitable for pill masses, etc.

ARTICLES DISMISSED FROM THE PHARMACOPŒIA.

ABSTRACTS.—Introduced in the 1880 U. S. P. as substitutes for powdered extracts, having the advantage of unchanging condition, and being of uniform strength in proportion to the crude drug; they were never generally adopted for use, and their omission now affords a good precedent for not repeating a similar attempt to push a drug into use, through the Pharmacopœia, for which no demand exists. The Pharmacopœia should record and describe staple drugs, but not seek to introduce new products; its province is to supply, not to create, demand.

ÆTHER.—The 1880 U. S. P. made two strengths of ether official: 74 and 94 per cent., termed respectively Æther and Æther Fortior. The 74 per cent. has been dropped, and the stronger ether—increased to 96 per cent.—is alone official under the simple term Æther (Ether). The expediency of this change to a single standard cannot be questioned; it will insure more accurate results from the use of this anæsthetic and general solvent, which heretofore have often been disappointing because of unapproved interchange of weaker and stronger ethers.

CHLOROFORMUM VENALE.—Of the two grades in the 1889 T. S. P. the weaker has been eliminated,—just as with ether, and the same remarks on the advantage of a single standard apply here. Pure chloroform is now readily prepared from acetone, and cheaply enough—which was not the case ten years ago—so that the necessity for the commercial product no longer exists. A strict interpretation of the rule which excludes products by patented processes would have prevented retaining chloroform in the U. S. P., because every pound of chloroform used in this country now is made by a patented process; the Committee undoubtedly knew this, but decided to include the product on the specious excuse that chloroform can be produced by a free process, even if it is not.

CINCHONA.—The old standard specified three kinds: *Cinchona*, bark of any species of cinchona, containing at least 3 per cent. of total alkaloids; *Cinchona Flava*, bark of trunk of Cinchona Calisaya, containing at least 2 per cent. of quinine; and *Cinchona Rubra*, bark of trunk of Cinchona Succirubra, containing at least 2 per cent. quinine.

Of these the term Cinchona Flava has been dropped, and the kinds retained are described as:

*Cinchona*, the bark of C. Calisaya and other species, containing at least 5 per cent. total alkaloids and 2.5 per cent. quinine; and

*Cinchona Rubra*, bark of C. Succirubra, containing not less than 5 per cent. total alkaloids.

This is following the practice of commerce, which long ago dropped the ordinary yellow, loxa, and other inferior varieties; the red Cinchona bark, so-called "Peruvian," transplanted to and extensively cultivated in Java and elsewhere, is usually of at least double the percentage requirement of the new 1890 U. S. P., and is the staple cinchona of the market.

The process of assay for alkaloids of cinchona, in con formity with the new usage, is according to the volumetric method, which is much more convenient and generally accurate than the former gravimetric process.

FEL BOVIS.—The inspissated ox-gall has been dropped, and the purified remains unchanged. This remedy has lately been taken up again with some enthusiasm by American therapists, and having only a single and uniform purified preparation available, the results will be more uniform and satisfactory.

GAULTHERIA has been dropped, but—rather inconsistently—the oleum gaultheria is retained, and its substitutes, methyl salicylate and oil of betula, are also introduced as official. As a matter of fact, the true oil of gaultheria is practically unobtainable and out of the market, and methyl salicylate is now currently supplied in 99 cases out of 100; consequently it might just as well have been omitted from the Pharmacopœia. And while economizing by eliminating useless and "dead-letter" products, the oil of Betula should have been kept out also.

GUTTA-PERCHA.—This product and its solution have been dropped, and no substitute offered. Gutta-percha solution is a serviceable preparation for the surgeon, and will endure with undiminished reputation its dismissal from the U. S. P.; perhaps, too, it will enter equally good company in the new National Formulary, or even by associating with the ex-communicated new remedies.

SODII BICARBONAS VENALIS.—The 1880 U. S. P. contained two grades: pure and commercial, corresponding respectively to 99 and 95 per cent. of the pure salt; in the new U. S. P. only one grade is called for, requiring 98.6 per cent. of the pure salt. This is a commendable change.

Eighty odd other members of the 1880 U. S. P. have been dropped, but all of so little prominence as to merit no special comment.

### ADDITIONS TO THE PHARMACOPŒIA.

Of articles added to the U. S. P. the following seem worth special notice.

ACETANILID.—This is the only member of the "new remedy era" antipyretics admitted to the hallowed pages of the U. S. P. 1890. Individually it deserves preferment, but how lonely it must feel in its exalted place, and with all its comrades, antipyrin, chloralamid, phenacetin, phenocoll, sulfonal, etc., "left out in the cold."

ACIDUM STEARICUM.—Has been introduced solely for use in making glycerin suppositories.

ALCOHOL.—Besides the "94 per cent." and the "dilute," the "absolute" and a "deodorized" alcohol have been added. The latter is used in preparing some of the official spirits, and merits preference because furnishing better preparations of their kind. The dilute is prepared differently and of different percentage than in the last U. S. P., which prescribed equal parts by weight of alcohol (94 per cent.) and water, giving a 45.5 per cent. by weight alcohol; the new U. S. P. directs equal parts by volume, and furnishes a 41 per cent. by weight alcohol. Inasmuch as it has been almost unexceptionally the custom—contrary to the official directions of the 1880 U. S. P.—to measure the alcohol and water, the new directions simply follow and authorize common practice.

ALOE BARBADENSIS.—Has been added probably in deference to the fact that it is official in the British Pharmacopœia, and is used largely here; the Socotrine Aloes is retained, and is preferred in the official preparations of the U. S. P.

ALOIN.—This active principle of aloes has come into extensive use of late years, recommending itself principally because it is a simple body, from which exact and reliable therapeutic effect can be obtained, and hence is preferable to the bulky and variable crude aloes.

AQUA AURANTII FLORUM.—The 1880 U. S. P. directed the preparation by distillation from fresh flowers; this, of course, was impracticable for the druggist, and it has been customary to purchase the French triple orange flower water and dilute, or to prepare it from the oil. The 1890 U. S. P. drops both orange flowers and the distilling process, and adopts the triple orange flower water as *aq. aurant. flor. fortior*, and directs the mixture of equal parts of this and water to prepare the regular *aq. aurant. flor.* for use in syrups and other preparations.—*Notes on New Remedies.*

